# Neuroinvasive *Listeria monocytogenes* infection triggers accumulation of brain CD8^+^ tissue-resident memory T cells in a miR-155-dependent fashion

**DOI:** 10.1186/s12974-020-01929-8

**Published:** 2020-09-02

**Authors:** Benjamin R. Cassidy, Miao Zhang, William E. Sonntag, Douglas A. Drevets

**Affiliations:** 1 Department of Medicine, University of Oklahoma Health Sciences Center, Oklahoma City, OK USA; 2 Department of Biochemistry & Molecular Biology, University of Oklahoma Health Sciences Center, Oklahoma City, OK USA; 3 Department of Veterans Affairs Medical Center, Oklahoma City, OK USA; 4 Section of Infectious Diseases, 800 Stanton L. Young, Suite 7300, Oklahoma City, OK 73104 USA

**Keywords:** miR-155, Tissue-resident memory cells, *Listeria*, Meningitis, Sepsis

## Abstract

**Background:**

Brain inflammation is a key cause of cognitive decline after central nervous system (CNS) infections. A thorough understanding of immune responses to CNS infection is essential for developing anti-inflammatory interventions that improve outcomes. Tissue-resident memory T cells (*T*_RM_) are non-recirculating memory T cells that provide surveillance of previously infected tissues. However, in addition to protecting the brain against reinfection, brain *T*_RM_ can contribute to post-infectious neuroinflammation. We hypothesized that accumulation of CD8^+^ *T*_RM_ in the brain could be reduced by inhibiting microRNA (miR)-155, a microRNA that influences development of cytotoxic CD8^+^ T lymphocytes during infection.

**Methods:**

C57BL/6J mice were infected by intraperitoneal injection with a lethal inoculum of *Listeria monocytogenes* (*Lm*) then treated with antibiotics. Flow cytometry was used to quantify specific populations of brain leukocytes 28–29 days (d) post-infection (p.i.). To test the degree to which miR-155 altered leukocyte influxes into the brain, infected mice were injected with a miR-155 inhibitor or locked nucleic acid (LNA) scramble control 2d, 4d, 6d, and 8d p.i. along with antibiotic treatment. Bacterial loads in spleen and liver and body weights were measured up to 7d p.i. Brain leukocytes were analyzed 14d and 28d p.i. Confirmatory studies were performed in mutated mice lacking miR-155 (miR-155^−/−^)

**Results:**

*Lm* infection significantly increased the numbers of brain CD3^+^CD8^+^ lymphocytes at 28d p.i. These cells were extravascular, and displayed markers characteristic of *T*_RM_, with the predominant phenotype of CD44^+^CD62L^-^CD69^+^CX3CR1^−^. Further analysis showed that > 75% of brain *T*_RM_ also expressed CD49a, PD-1, Ly6C, CD103, and CD127. Mice injected with miR-155 inhibitor lost less weight through 7d p.i. than did control mice, whereas bacterial loads in brain, liver, and spleen were not different from controls. By 28d p.i., the numbers of brain CD8^+^ T_RM_ cells were significantly decreased in mice treated with the inhibitor compared with controls. Similarly, miR-155^−/−^ mice showed significantly reduced numbers of brain CD8^+^ *T*_RM_ cells by 28d p.i.

**Conclusions:**

Brain CD8^+^ T_RM_ populations are established during neuroinvasive *Lm* infection. Accumulation of brain CD8^+^ T_RM_ cells is reduced by blocking miR-155 and in miR-155^−/−^ mice, indicating that this molecule has a critical role in development of these specialized cells. Administering anti-miR-155 during infection could provide a novel avenue for reducing post-infectious neuroinflammation.

## Background

Infectious diseases of the central nervous system (CNS) are significant causes of morbidity and mortality throughput the world. In this regard, meningitis and encephalitis rank as the 4th and 10th largest contributors, respectively, to age-standardized disability-adjusted life-years among all neurological disorders [1]. Although antibiotics notably improve case-fatality rates for bacterial CNS infections, a substantial minority of patients, particularly adults, still succumb to the infection and 12–35% of survivors have residual neurological/cognitive dysfunction [2, 3]. Moreover, adjunctive anti-inflammatory agents such as corticosteroids have limited applicability as they do not improve immediate outcomes in all populations, nor do they improve long-term cognitive outcomes [4–6]. Given the key role of inflammation in infection-induced neurological injury, there is a pressing need to understand host responses that persist after resolution of infection as a potential venue for developing adjunctive therapies that can improve outcomes to CNS infections.

Leukocytes recruited to the infected CNS are a key source of pro-inflammatory mediators, such as IFN-γ and TNF, as well as chemokines that recruit additional cells and amplify the inflammatory response. These pro-inflammatory mediators activate microglia and facilitate pathogen removal, but also negatively affect cognitive function and can cause mood disorders in survivors [7]. Most populations of bone marrow-derived leukocytes recruited into infected tissues, including the brain, return to their pre-infection size after the infection is cleared [8]. However, a recently described population of T lymphocytes, T resident memory cells (*T*_RM_), establish long-lived populations within infected tissues to provide immune surveillance and rapid pathogen removal in the case of re-infection [9]. *T*_RM_ are found within the brain following viral and parasitic infection and are necessary to contain chronic pathogen replication [10–12], and have recently been studied in the context of bacterial infection [13]. Prior studies demonstrated that brain *T*_RM_ (BT_RM_) promoted rapid clearance of ovalbumin-expressing *Listeria monocytogenes* (*Lm*) that were inoculated intracranially into mice previously injected with ovalbumin-pulsed dendritic cells [11]. Recent studies show that BT_RM_ can also be generated during peripheral viral and bacterial infections, and promote pathogen clearance from the brain [13].

Despite this clearly documented role in protection against reinfection, it is possible that CD8^+^ BT_RM_ also contribute to chronic CNS inflammation. For example, virally induced CD8^+^ BT_RM_ have been shown to trigger cognitive decline, primarily via IFN-γ stimulation of microglia [14]. Similarly, these cells are thought to contribute to gliosis in chronic HIV infection of the brain [15]. Other CD8^+^ T cells found in the brains of aged mice have been shown to inhibit proliferation of neural stem cells, also via expression of IFN-γ [16].Thus, it is possible that BT_RM_ induced during bacterial infection can contribute to chronic neuroinflammation after infection.

Experimental *Lm* infection of mice is a well-studied model of infection by a facultative intracellular pathogen and has been applied to the study of CNS infection [17]. In this model, bacteria that are injected intravenously (i.v.) are rapidly removed from the bloodstream by the liver and spleen and do not directly invade the brain [18–20]. However, given a sufficient inoculum, bacteria injected i.v. or intraperitoneally (i.p.) overcome host defenses in the liver and spleen, then spread to the bone marrow and then invade the brain via parasitized Ly6C^hi^ monocytes in a secondary bacteremia [21, 22]. To study brain inflammation in a scenario similar to that of patients with CNS infection, we induced neuroinvasive *Lm* infection in mice via systemic injection, then administered the same antibiotic used for human infection to the infected animals [23]. In this model, as also in humans with bacterial CNS infection [24], antibiotic treatment is necessary for the animals to survive the infection thereby allowing investigation of BT_RM_ accumulation and the molecular mechanisms that undergird this process.

These experiments showed that neuroinvasive CNS infection triggered large influxes of bone marrow-derived leukocytes comprised predominately of activated CD3^+^ T lymphocytes, and which also included neutrophils and Ly6C^hi^ monocytes. Interestingly, the numbers of activated lymphocytes remained elevated in the brain at least 14 days (d) post-infection (p.i.), approximately 7 days after pathogen elimination, and were significantly lower in mice lacking microRNA-155 (miR-155) compared to normal mice. miR-155 is a non-coding RNA that is produced by lymphoid cells, myeloid cells, and bone marrow, and influences diverse aspects of hematopoiesis and the inflammatory response [25–28]. microRNAs alter expression of their targets by binding to complementary sites on target mRNA, which inhibits translation and/or promotes degradation of the target mRNA [29, 30]. Due to the diversity of targets, expression of miR-155 can result in either pro-inflammatory or anti-inflammatory effects, depending upon the cell or condition [31]. During infection, miR-155 is upregulated via NFκB in response to TLR-signaling and cytokines such as IFN-β, IFN-γ, and TNF, and alters inflammation by multiple mechanisms including modulating TLR signaling, transcription factor expression, and cytokine production [28, 32] miR-155 is also necessary for optimal development of cytokine-secreting CD8^+^ T lymphocytes during *Lm* infection [33, 34], as well as other infections, e.g., Herpes simplex virus [34, 35]. Notably, recent data suggests that miR-155 is also critical in the development of *T*_RM_, likely via miR-155 modulation of the transcription factor T-bet, which is critical for CD8^+^ *T*_RM_ development [36, 37]. Thus, we hypothesized that accumulation of infection-induced BT_RM_ could be reduced by inhibiting miR-155.

In this series of studies, we demonstrate that neuroinvasive *Lm* infection after peripheral inoculation induces a population of CD8^+^ T lymphocytes bearing the phenotype of BT_RM_. Additionally, we show that numbers of CD8^+^ BT_RM_ are significantly reduced by peripheral injection of a miR-155 inhibitor molecule given along with antibiotics, as well as in mutated mice lacking miR-155. These results demonstrate that significant numbers of *T*_RM_ reside in the brain after resolution of neuroinvasive bacterial infection and that miR-155 is required for their development. These results suggest that miR-155 inhibition could be a novel adjunctive treatment regimen during bacterial CNS infection, and could lessen the associated long-term inflammation.

## Materials and methods

### Antibodies

Fluorochrome-conjugated mAb directed against specific antigens and isotype-matched control antibodies were purchased from BD Pharmingen (San Diego, CA): CD62L (clone MEL-14, fluorophore BV510), BioLegend (San Diego, CA): CD11b (M1/70, BV421), CD3 (17A2, PE), CD8a (53-6.7, Alexa Fluor 488), CD4 (RM4-5/GK1.5, BV605 and BV785), CD44 (IM7, PerCP/Cy5.5), CD45 (30-F11, PE/Cy7 and PE/Dazzle™), CX3CR1 (SA011F11, BV605), Ly-6G (1A8, BV510), Ly-6C (HK1.4, PerCP/Cy5.5), CD69 (H1.2F3, BV711), CD103 (2E7, APC), PD-1 (29F.1A12, PE/Cy7), CD49a (HMα1, APC), and CD127 (A7R34, BV421).

### Bacteria

*Lm* strain EGD was originally obtained from P.A. Campbell [38]. Bacteria were stored in brain-heart infusion (BHI) broth (Difco, Detroit, MI) at 10^9^ CFU/mL at − 80 °C. For experiments, the stock culture was diluted 1:10,000 into BHI and cultured overnight at 37 °C with shaking.

### Animal infection and antibiotic treatment

This study was carried out with approval from the Institutional Animal Care and use Committee (IACUC) of the University of Oklahoma HSC (OUHSC). All animals were purchased from Jackson Laboratories (Bar Harbor, ME). Male and female C57BL/6J and age- and sex-matched B6. Cg-*Mir155*^*tm1.1Rsky*^/J (miR-155^−/−^) mice 8–12 weeks of age were used in experiments as indicated. Mice were infected i.p. with 500 μL PBS containing 1.2–2.4 × 10^5^ CFU *L*. *monocytogenes* then were treated with antibiotics as previously described [23]. Mice were never sedated for i.p. injections. Infection was performed i.p. because in our hands it is more time efficient, requires less animal manipulation and restraint, and has a lower failure rate. Mice were weighed daily for 7d. Infected and uninfected mice were injected i.p. with 2 mg ampicillin (Butler Schein Animal Health, Dublin, OH) three times at 10–12-h intervals beginning 48 h p.i. Bubblegum-flavored amoxicillin (2 mg/mL final concentration) was added to the drinking water 3d p.i. and continued until sacrifice or 14d p.i., as indicated. Some uninfected mice received three doses of i.p. ampicillin plus oral amoxicillin as described above to control for antibiotic effects, and are indicated as such. Mice were euthanized by CO_2_ asphyxiation, exsanguinated via femoral vein cut-down, and perfused transcardially with 25 mL iced, sterile PBS containing 2 U/mL heparin. Brain, spleen, and liver were removed aseptically after perfusion during necropsy. Blood was not collected. Organs used for culture were weighed, then were homogenized in sterile ddH_2_O. Serial 10-fold dilutions were plated on tryptic soy agar and incubated at 37 °C for 24 h. CFU *Lm* were quantified the following day.

miRCURY Locked Nucleic Acid™ (LNA) miRNA inhibitor oligonucleotide was custom ordered from QIAGEN (Hilden, Germany). miR-155 inhibitor (Product MMU-MIR-155-5P INH, Cat. No. 339203 YCI0200322-FZA, sequence 5′-3′ TCACAATTAGCATTA) and negative control A “LNA scramble” (Product NEGATIVE CONTROL A, Cat. No. 339203 YCI0200319-FZA, sequence 5′-3′ ACGTCTATACGCCCA) were used according to the manufacturer’s guidelines. Each oligonucleotide was resuspended in 1× PBS to a final concentration of 2 mg/mL, aliquoted, and frozen at − 80 °C until use. Prior to injection, each molecule was dissolved in 1× PBS at a concentration of 2 mg/mL. They were dosed at 20 mg/kg body weight injected subcutaneously (s.c.) in the flank on 2d, 4d, 6d, and 8d p.i. Injections were alternated between the left and right flanks.

### Tissue preparation and magnetic cell sorting

Perfused brains were harvested then enzymatically digested for 45 min at 37 °C in Miltenyi C tubes (Miltenyi Biotec, San Diego, CA) containing 0.5 mg/mL Collagenase IV and 0.025 mg/mL DNAse I in RPMI-1640 (ATCC, Manassas, VA) plus 1% penicillin/streptomycin and 10% fetal bovine serum. The slurry was passed through a 70 nm cell strainer with 10 mL HBSS without Ca/Mg (Lonza, Basel, Switzerland) and centrifuged at 300×*g* for 10 min at room temperature. The supernatant was discarded, and the cells were suspended in 30% Percoll (GE Healthcare, Chicago, IL) in a 15 mL conical tube and centrifuged at 700×*g* at RT for 10 min to remove myelin. The cell pellet was washed once with PBS + 0.5% BSA at 300×*g* for 10 min at 4 °C then erythrocytes were lysed by incubation for 5 min at RT using RBC Lysis Buffer (Life Technologies Corp., Carlsbad, CA). The leukocytes were washed twice with PBS + 0.5% BSA at 300×*g* for 10 min at 4 °C then resuspended in 3 mL of PBS + 0.5% BSA for counting (Countess II FL Automated Cell Counter, Life Technologies Corp.). In some experiments, specific cells were collected by magnetic sorting using CD11b Microglia Microbeads or CD45 Microbeads (Miltenyi) and LS columns according to the manufacturer’s protocol.

### Flow cytometry

Cells were incubated on ice for 30 min with 2 μL of anti-CD16/32 TruStain fcX (BioLegend, San Diego, CA) plus 10 μL of Brilliant Stain Buffer Plus (BD Biosciences, Franklin Lakes, NJ). Next, fluorochrome-labeled mAb were added, and the cells were incubated at RT in the dark for 30 min then were washed twice with 3 mL FACS buffer (PBS + 0.5% BSA + 0.1% NaN_3_). Cells were post-fixed with 200 μL IC Fixation buffer (Life Technologies Corp.) for 30 min at RT in the dark, then washed again with 3 mL FACS buffer, and stored at 4 °C in the dark until analyzed. Flow cytometry was performed on a Stratedigm S1200Ex (Stratedigm Inc, San Jose, CA) and analyzed with CellCapTure software (Stratedigm).

Intravascular leukocytes were distinguished from those located in brain parenchyma (extravascular) as previously described [39]. Briefly, mice were anesthetized with isoflurane then injected i.v. into the retroorbital sinus with 3 μg PE/Dazzle™-labeled anti-CD45 mAb (30-F11) in 50 μL of sterile 1× Dulbecco’s phosphate-buffered saline (DPBS). After 5 min, the mouse was euthanized by CO_2_ asphyxiation and exsanguinated. After tissue preparation, leukocytes were incubated with PE-Cy7-labeled CD45 mAb, as well as other markers. Using this technique, CD45^+^ cells bearing PE/Dazzle were considered intravascular, whereas those not bearing this label were deemed extravascular.

### Analysis of gene expression in specific populations of brain cells

*Lm* infected mice treated with miR-155 inhibitor or scramble were sacrificed 9d p.i. Brains cells from 2 mice were pooled together. CD45^hi^ brain cells were collected by magnetic sorting and sorted again using a FACSAria Fusion (BD Biosciences) cell sorter. The cells were then lysed in Qiazol Lysis Reagent, and total RNA was extracted using a miRNeasy Micro Kit (QIAGEN, Redwood City, CA) according to the manufacturer’s instructions. mRNA expression analysis using the NanoString nCounter® Immunology Panel (Mouse) was performed according to manufacturer’s protocol (NanoString Technologies Inc., Seattle, WA). nCounts were normalized to the geometric mean of the internal positive controls using nSolver™ Analysis Software (NanoString Technologies Inc., Seattle, WA). Results shown are the mean ± SD nCounts from 3 cell pools/time point.

### Quantitative real-time PCR

RNA was extracted from mouse tissue using a standard protocol applying the direct-zol RNA Miniprep kit (Zymo Research, Irvine, CA) according to the manufacturer’s instructions. To determine miRNA expression, TaqMan microRNA reverse transcription kit and TaqMan miRNA assay kit from Life Technologies were used following the manufacturer’s protocol. Primers for miR-155 (assay ID 002571), and snoRNA 135 (assay ID 001230) were purchased from Invitrogen (Carlsbad, CA) and used in standard TaqMan assays. The expression of miRNA was normalized to expression of a stable endogenous control gene (snoRNA-135) within the same sample to account for variation in sample loading. The ddCt method [40] was used to estimate relative changes in miRNA expression.

### Statistical analysis

Data analysis was performed using the GraphPad Prism 8 statistical suite. Data were analyzed using a two-tailed Student’s *t* test assuming equal variance or the Mann-Whitney *U* test to compare two groups. For each of these, *p* < 0.05 was considered significant. Statistical analysis of results in Fig. 3 was performed using one-way ANOVA without the assumption of equal variance (Brown-Forsythe and Welch’s ANOVA tests) comparing results from each group to the uninfected (control) group followed by a post-hoc comparison using Dunnett’s T3 multiple comparisons test. Results in Fig. 6 comparing the interaction between infection status and miR-155 expression were compared using a two-way ANOVA followed by Tukey’s multiple comparisons test.

## Results

### Neuroinvasive *Lm* infection leads to increased numbers of CD8^+^*T*_RM_ in the brain at 29d p.i.

**Fig. 1:**
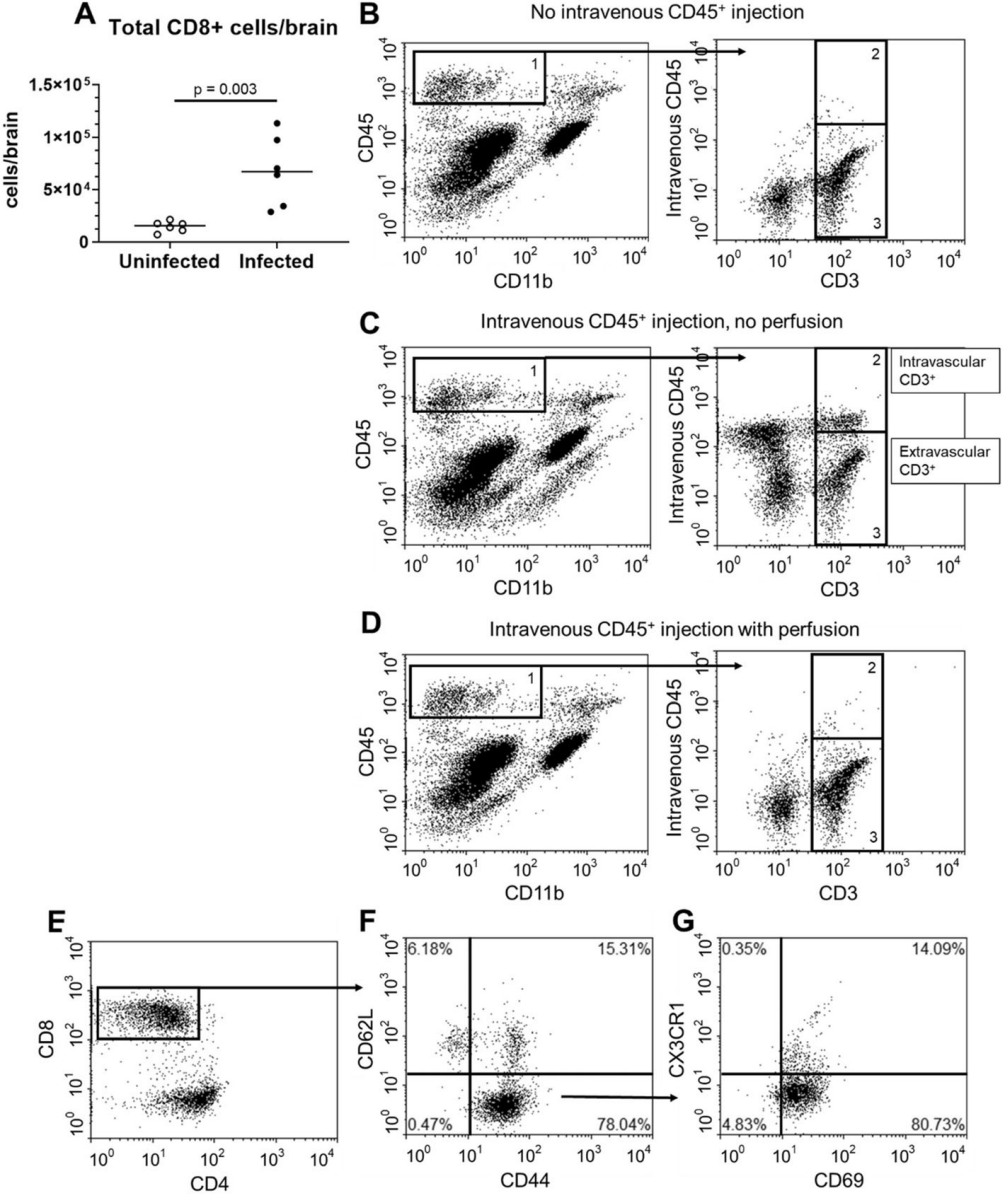
Neuroinvasive *Lm* infection induces accumulation of brain CD8^+^ T_RM_ cells. **a** Male C57BL/6J mice (*n* = 9) were infected with 2.0 × 10^5^ CFU *Lm* EGD and treated with antibiotics. Uninfected mice (*n* = 4) were injected i.p. with PBS then also received antibiotics. Mice were euthanized 29d p.i., and brain leukocytes were quantified by FACS. Symbols represent individual mice. Significance was calculated via two-tailed Student’s *t* test. **b–d** At 29d p.i., mice were/were not injected i.v. with CD45-PE/Dazzle 10 min prior to euthanasia as indicated. For this proof-of-concept experiment, some brains were harvested without perfusion prior to enzymatic digestion and FACS analysis of leukocytes. TheCD45^hi^CD11b^neg-low^ population **(**box 1) was further gated on CD3^+^ to identify T lymphocytes. Discrimination between intravascular and extravascular cells was determined by staining with an isotype control. **b** Representative dot plot of a mouse that did not receive intravenous CD45, used as a negative control. **c** Representative dot plot of an unperfused mouse that received i.v. PE/Dazzle. CD3^+^ cells stained by PE/Dazzle were considered intravascular (gate 2), and CD3^+^ cells stained by PE/Dazzle were considered extravascular (gate 3). **d** Representative dot plot of a perfused mouse that received i.v. PE/Dazzle. After perfusion, 97.5 ± 0.7% of CD3^+^ cells were unstained, indicating they were extravascular (mean ± SD, *n* = 5). **e** Representative dot plots of extravascular CD3^+^ cells in a mouse that received i.v. PE/Dazzle. Extravascular CD3^+^ cells were gated on CD8^+^. These cells were predominately CD44^+^CD62L^−^ (**f**), and CD69^+^CX3CR1^−^ (**g**)

During acute neuroinvasive *Lm* infection, bone marrow-derived leukocytes, including neutrophils, monocytes, and CD4^+^ and CD8^+^ T lymphocytes, are recruited into the brain [23]. We tested the extent to which CD8^+^ T lymphocytes remain in the brain after resolution of infection and determined if *T*_RM_ cells were contained within this population. For this, C57BL/6J mice were infected with 2.0 × 10^5^ CFU *Lm* and treated with antibiotics as previously described [23]. This infection model reliably induces brain infection 2–3d post-inoculation, accompanied by influxes of myeloid cells and lymphocytes, with bacterial sterilization achieved by 7d p.i. In experiments described here, mice were sacrificed 29d p.i. and their brain leukocytes were analyzed by FACS. Results in Fig. 1 show *Lm* infected mice had greater than 4-fold more CD3^+^CD8^+^ T lymphocytes (68,100 ± 33,600, mean ± SD, *n* = 6) than did uninfected mice (14,800 ± 5100, *n* = 6).

**Fig. 2.**
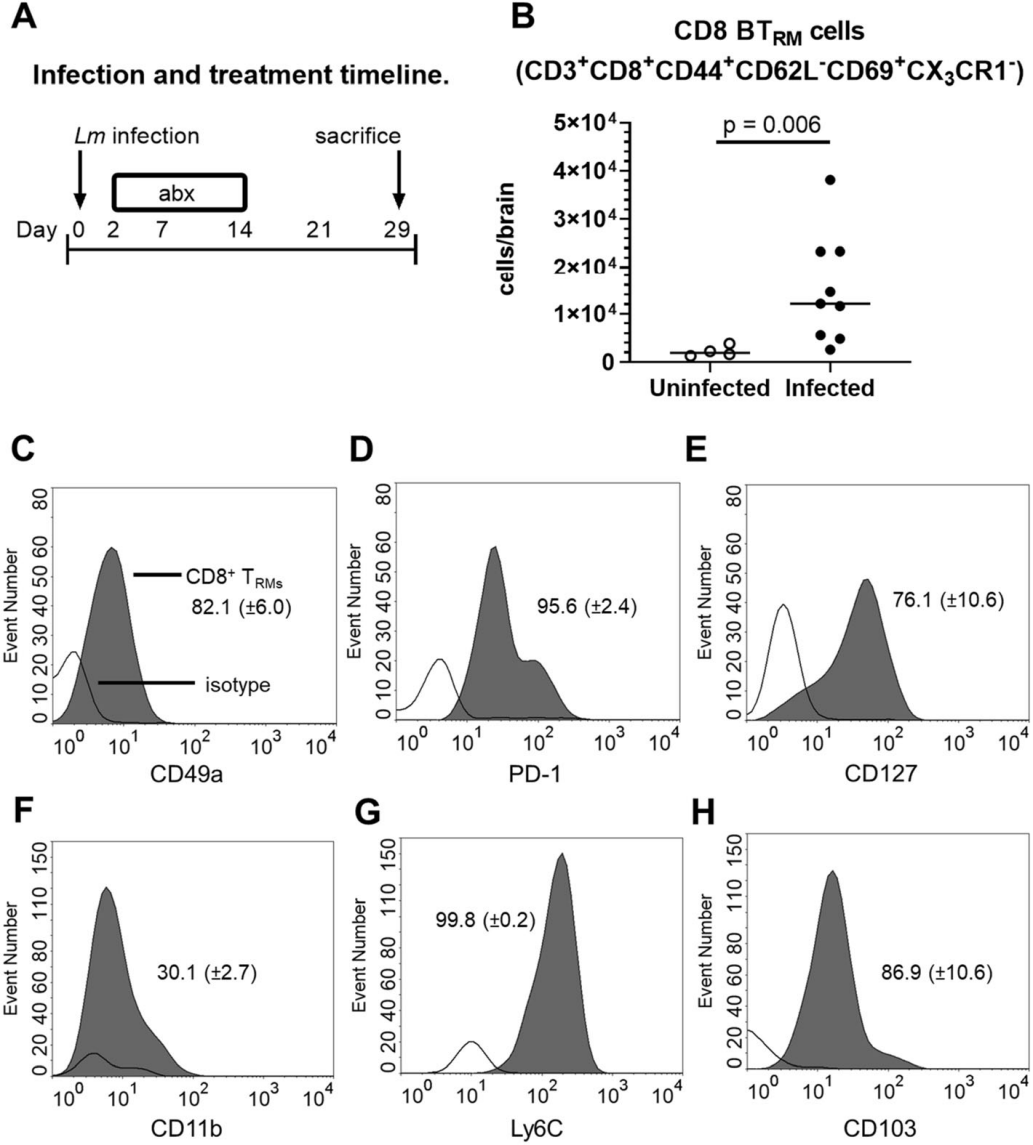
*Lm* infection significantly increases CD8^+^ BT_RM_ populations at d29 p.i. Male C57BL/6J mice were infected with 2.0 × 10^5^ CFU *Lm* EGD and treated with antibiotics. Control mice were uninfected, but treated with antibiotics. Mice were euthanized 29d p.i. and brains were harvested. **a** Cell populations were measured by FACS. **b** Each symbol represents an individual mouse, p value calculated by Mann-Whitney *U* test. **c**–**h** Histograms from representative mice show distributions of isotype-stained cells (no fill) and gated CD8^+^ *T*_RM_ (gray fill). Mean (± SD, n 4–9) percent marker positive cells are given

To determine the degree to which this cell population was contained within the vascular system or were extravascular, mice were injected i.v. with PE/Dazzle™-labeled anti-CD45 mAb prior to brain removal and subsequent incubation with anti-CD45 conjugated to a different fluorochrome. Differential labeling of CD45^hi^ cells as detected by FACS was used to discriminate intravascular (PE/Dazzle^+^) from extravascular (PE/Dazzle^-^) cells [39]. Extravascular CD8^+^ cells were predominantly CD44^+^CD62L^−^CD69^+^CX3CR1^−^ (Fig. 2b) consistent with the phenotype of BT_RM_ reported in other infection models [13, 41]. Infected mice had nearly seven-fold more *T*_RM_ at 29d p.i. (15,000 ± 11,000 mean ± SD, *n* = 8) than did uninfected mice (2300 ± 1100, *n* = 4, *p* = 0.006) (Fig. 2b). Notably, CD3^+^CD8^+^CD44^+^CD62L^−^CD69^+^CX3CR1^−^ cells were uniformly not labeled by anti-CD45 injected i.v. Thus, CD3^+^CD8^+^CD44^+^CD62L^−^CD69^+^CX3CR1^−^ cells were considered extravascular BT_RM_. Further analysis of BT_RM_ cells showed they expressed a CD49a^+^PD-1^+^Ly6C^+^CD103^+^ phenotype (Fig. 2c–h). These markers were studied because CD49, PD-1, and CD103 are reported to be expressed on brain *T*_RM_ [41], and Ly-6C and CD11b are markers of T cell activation [42, 43]. In addition, most cells expressed CD127 and a lesser proportion expressed low amounts of CD11b. Taken together, these results indicate that neuroinvasive *Lm* infection following systemic inoculation of bacteria induces accumulation of CD8^+^ *T*_RM_ in the brain, which remain after antimicrobial treatment and sterilization of the infection.

### Systemically administered miR-155 inhibitor reduces accumulation of CD8^+^ BT_RM_

**Fig. 3:**
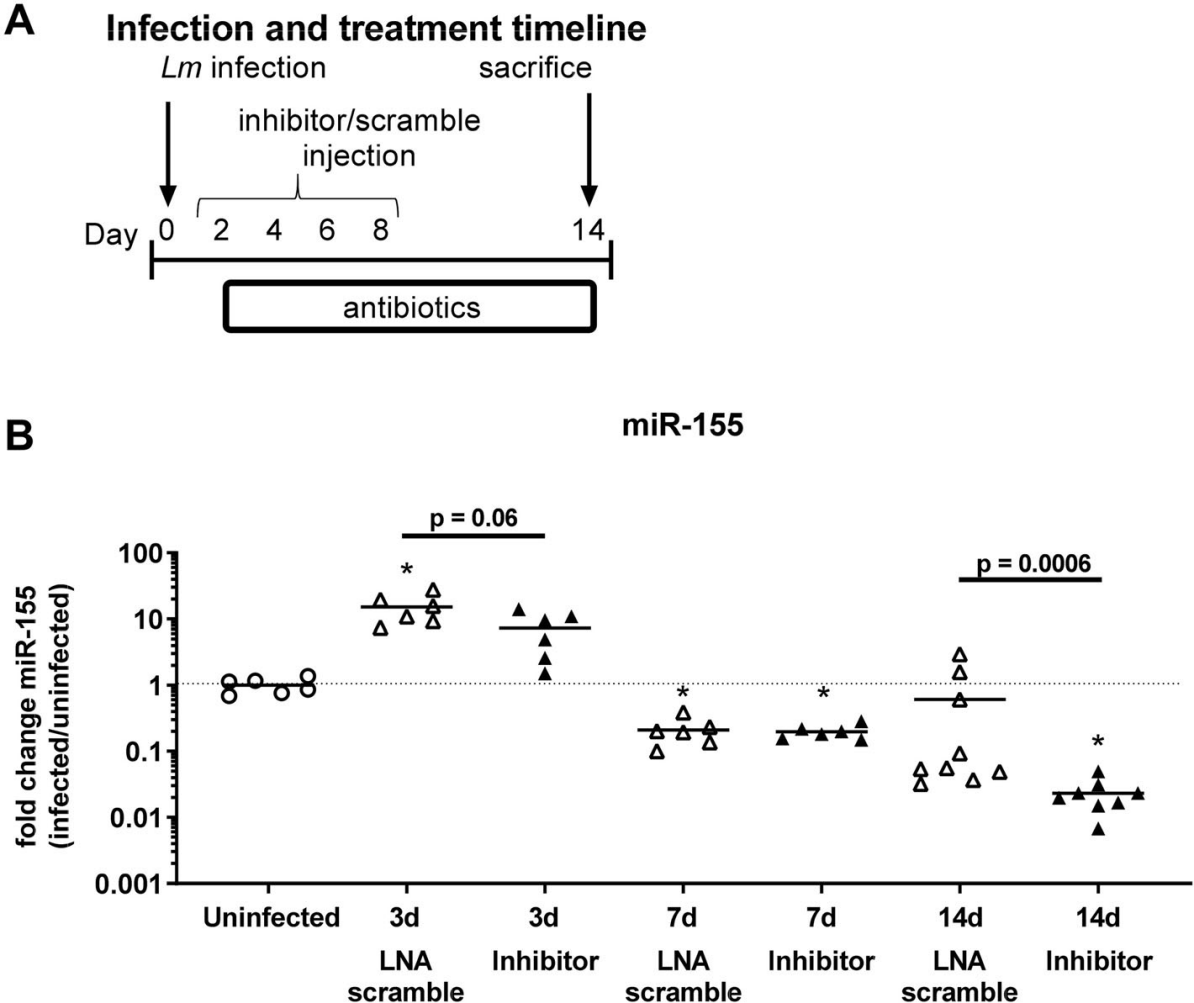
miR-155 inhibitor molecule inhibits expression of miR-155 in spleens of *Lm* infected mice. Male C57BL/6J mice were infected with 2.2–2.3 × 10^5^ CFU *Lm* EGD, then treated with antibiotics. Uninfected mice (circle) received antibiotics. miR-155 inhibitor (black upward-pointing triangle, black downward-pointing triangle) or LNA scramble (white upward-pointing triangle, white downward-pointing triangle) injected s.c. on 2d, 4d, 6d, and 8d p.i. Mice were sacrificed on 3d, 7d, 14d p.i. Experimental timeline is shown in (**a**). miR-155 expression in spleens was measured by qPCR and normalized to sno135 (**b**). Values are expressed in terms of fold change, with uninfected = 1. Significant *p* values compared with uninfected mice by one-way ANOVA with Dunnett’s T3 multiple comparisons test are indicated (*p < 0.05). Statistical differences between infected mice treated with miR-155 inhibitor or LNA scramble at individual time points calculated via two-tailed Student’s *t* test (3d) Mann-Whitney *U* test (d14) are given.

Results from others show that miR-155 is required for optimal development of cytotoxic CD8^+^-T lymphocyte response during systemic *Lm* infection [34, 44]. Additionally, work from our lab showed that miR-155^−/−^ mice have significantly reduced numbers of brain CD8^+^ cells 14d p.i [23]. Thus, we tested the degree to which accumulation of CD8^+^ BT_RM_ cells could be reduced by inhibition of miR-155. For this, mice were infected with *Lm* and treated with antibiotics plus miR-155 inhibitor, or antibiotics plus LNA scramble, injected 2d, 4d, 6d, and 8d p.i. (Fig. 3a). Expression of miR-155 in the spleen was measured by qPCR before infection, as well as 3d, 7d, and 14d p.i. Results in Fig. 3b show miR-155 expression increased significantly at 3d p.i. in LNA scramble-treated infected mice compared with uninfected animals, but not in inhibitor-treated infected mice. Mice treated with miR-155 inhibitor had a strong trend toward decreased miR-155 expression by 3d p.i. compared to LNA scramble-treated mice. By 7d p.i., miR-155 expression had decreased below pre-infection levels, and differences between miR-155 inhibitor- and LNA scramble-treated mice were not found. The pattern of miR-155 expression in the spleen, i.e., upregulation at 3d p.i. with a subsequent decline at 7d p.i., is similar to that observed in CD11b^+^ bone marrow cells in this model [23]. At 14d p.i., miR-155 expression in mice treated with miR-155 inhibitor remained significantly lower than uninfected mice and was also lower than in infected mice treated with LNA scramble. In addition to showing dynamic changes in miR-155 expression during and after acute infection, these results demonstrate that the inhibitor can reduce miR-155 expression in the spleen, and has the potential to modulate the immune response throughout the course of bacterial infection. However, its downstream effects are likely less robust than those observed in miR-155^−/−^ mice.

**Fig. 4:**
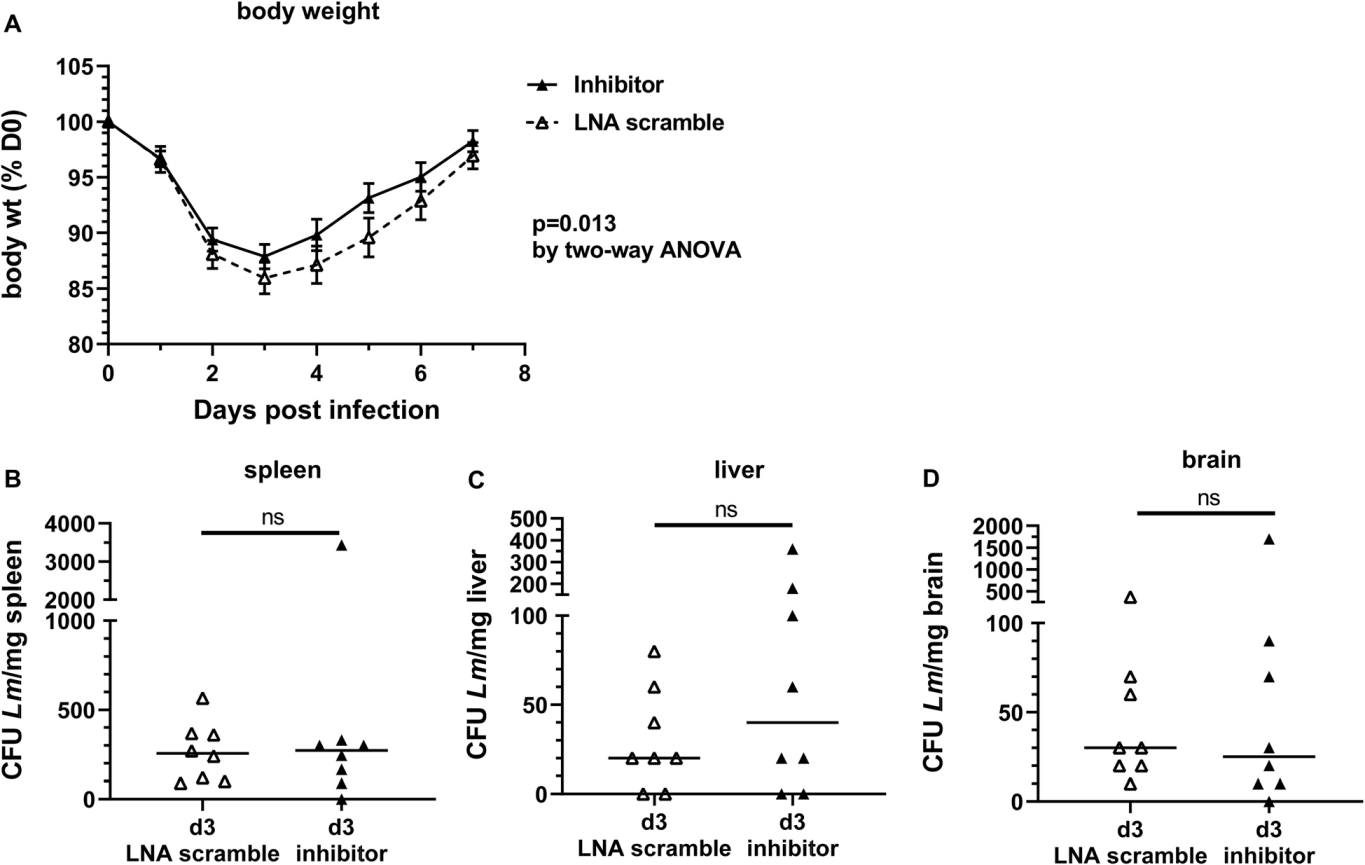
miR-155 inhibitor reduces infection-induced weight loss without altering bacterial load in liver and spleen. Male and female age-matched C57BL/6J mice were infected with 1.8–2.4 × 10^5^ CFU *Lm* EGD and treated with antibiotics beginning 2d p.i. miR-155 inhibitor or LNA scramble was injected s.c. on 2d, 4d, and 6d p.i. **a** Animals were weighed daily. Weights are expressed as a percentage of initial pre-infection weight for each mouse individually. *n* = 8–18 animals for each time point and treatment. Significance was determined via two-way ANOVA. **b–d** Organs were removed aseptically at necropsy, then weighed and homogenized in sterile dH_2_O. Bacterial loads in spleen (**b**), liver (**c**), and brain (**d**) at 3d p.i. were measured by serial dilution and plating on agar. Significance was determined via two-tailed *t* test

To gauge the effect of the miR-155 inhibitor on key physiologic and microbiologic parameters of systemic infection, mice were weighed daily through 7d p.i., and bacterial loads in spleen, liver, and brain were measured at 3d and 7d p.i. After infection, mice lost weight until d3 p.i., then steadily retuned to pre-infection weight, typically by 7d p.i. (Fig. 4a). During this time, mice treated with miR-155 inhibitor lost less weight than did LNA scramble-treated mice (*p* = 0.013, *n* = 8–18 per time point). Quantification of bacteria in spleens, livers, and brains revealed no significant differences between inhibitor- and LNA-scramble treated mice in either tissue at 3d p.i. (Fig. 4b–d). In addition, each of these tissues were cultured on 7d p.i. and found to be sterile in both groups of mice (data not shown). Our previous work in this model showed that miR-155^−/−^ mice had significantly fewer CFU *L*. *monocytogenes* in the liver, but not in the spleen or brain, at 3d p.i. than did normal mice, and that all organs in both groups were sterile by 7d p.i [18]. These data are consistent with the notion that the miR-155 inhibitor produces less robust changes than are observed in knock-out mice. They also indicate that differential bacterial infection is not driving downstream differences in brain inflammation between miR-155 treated and LNS scramble-treated mice.

### miR-155 inhibitor decreases expression of T cell associated genes in brain CD45^hi^ cells

Next, we tested the degree to which miR-155 inhibitor altered gene expression in brain leukocytes. Male mice were infected with 2.2 × 10^5^ CFU *Lm*, treated with antibiotics and miR-155 inhibitor or LNA scramble 2d, 4d, 6d, and 8d p.i., then harvested at 9d p.i. This particular time point in the infection was chosen as the animals would have received each of the injections of miR-155 inhibitor. Also, 9d p.i. is early enough in the infection that individual mice likely had sufficient numbers of bone marrow-derived cells in the brain, so that sufficient cell numbers for analysis of gene expression could be obtained from combining two mice per time point. CD45^+^ brain leukocytes were collected by magnetic sorting then were sorted by FACS into two populations: microglia (CD45^int^CD11b^hi^) and bone marrow-derived cells (CD45^hi^), and gene expression was analyzed by nCounts (Supplemental Fig. 1). Complete gene expression data sets are available in Supplemental data file 1.

Comparison of nCounts in CD45^+^ cells from miR-155 inhibitor and LNA scramble-treated mice identified 20 genes whose expression differed at a *p* ≤ 0.01 despite the small sample size of only three in each group (Table 1). Genes with reduced expression in miR-155 inhibitor-treated mice were critical for T lymphocyte development and function, e.g., *Cd8b1*, *Bcap31*, *Runx3*, and *Cd27* (Table 1) [45]. In contrast, several neutrophil and phagocyte-associated genes were markedly upregulated in the same samples, e.g., *S100a8*, *S100a9*, and *Camp*. FACS analysis of the cells used to measure gene expression showed no major population shifts between inhibitor-treated mice and scramble-treated mice at d9 p.i. (Supplemental Fig. 2). In contrast to findings in bone marrow-derived cells, analysis of gene expression in microglia showed no significant differences between miR-155 inhibitor and LNA scramble-treated mice (Supplemental data file 1). These data suggest peripherally administered miR-155 inhibitor has an effect on bone marrow-derived leukocytes entering the brain during infection, but does not directly affect microglia, likely because it does not cross the blood-brain barrier as suggested by the manufacturer’s product information [46].

**Table 1:** miR-155 inhibitor alters gene expression in CD45^hi^ cells isolated from brains d9 p.i.

Gene	LNA Scramble	miR-155 Inhibitor	Fold change
S100a9	1293 (1649)	22,090 (1944)	4.094
Camp	64 (45)	1027 (200)	3.994
S100a8	1307 (1609)	20,813 (3043)	3.993
Cxcr2	15 (9)	130 (28)	3.093
Il1r2	34 (19)	119 (23)	1.821
Plaur	141 (41)	473 (96)	1.748
Trem1	63 (17)	210 (51)	1.745
Csf3r	64 (19)	214 (52)	1.733
C3	76 (13)	187 (32)	1.297
Cd14	85 (14)	204 (35)	1.259
Itgam	185 (21)	379 (60)	1.035
Ccl6	62 (12)	125 (8)	1.011
Ifngr2	98 (21)	184 (16)	0.908
Tnf	238 (5)	205 (11)	− 0.211
Bcap31	660 (34)	550 (14)	− 0.261
Psmd7	300 (12)	238 (4)	− 0.332
Runx3	207 (12)	158 (2)	− 0.389
Nfatc2	352 (19)	268 (16)	− 0.394
Cd8b1	416 (14)	310 (34)	− 0.422
Cd27	370 (41)	261 (7)	− 0.507

Data are expressed as the mean (SD) nCounts, *n* = 3 cell pools/group. Displayed genes were significant to a *p* < 0.01 via 2-tailed Student’s *t* test. Fold change calculated as the log_2_ mean nCount (miR-155 inhibitor-treated /LNA scramble-treated)

### miR-155 is critical for *T*_RM_ accumulation in the brain post-infection

**Fig. 5:**
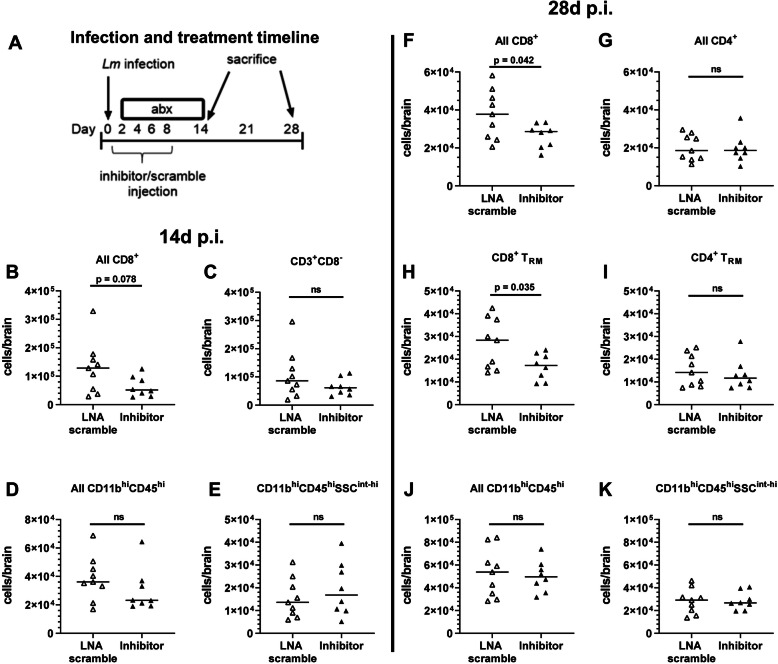
miR-155 is critical for CD8^+^ BT_RM_ accumulation in the CNS during neuroinvasive infection. Male (14d) or male and female (28d) C57BL/6J mice were infected with 2.0 × 10^5^ CFU *Lm* EGD, then treated with antibiotics beginning 2d p.i. **a** Mice were injected s.c. with miR-155 inhibitor (black upward-pointing triangle) or LNA scramble (white upward-pointing triangle) was injected s.c. on 2d, 4d, 6d, and 8d p.i. then sacrificed on 14d (**b–e**) or 28d (**f–k**) p.i. Brain leukocytes were analyzed by FACS. *n* = 8–9 (14d) or 4–5 (28d) for each sex and treatment. Symbols represent individual mice. Significance was calculated via two-tailed Student’s *t* test

Next, we tested the effect of peripherally injected miR-155 inhibitor on accumulation of CD8^+^ T lymphocytes and CD8^+^ BT_RM_ after infection. Male and female mice were infected, given antibiotics, and treated with miR-155 inhibitor or LNA scramble as described above, then brain leukocytes were analyzed by FACS at 14d and 28d p.i. At d14 p.i., there was a trend toward reduced numbers of brain CD8^+^ cells, but not in other populations measured (Fig. 5b–e). BT_RM_ were not specifically measured in these samples. However, by 28d p.i., numbers of CD8^+^ BT_RM_ and CD3^+^CD8^+^ cells were significantly decreased in miR-155 inhibitor-treated mice compared to LNA scramble-treated mice (Fig. 5f, h). There was a 30% decrease in all CD3^+^CD8^+^ cells in miR-155 inhibitor-treated mice compared to LNA scramble-treated mice (26,400 ± 6300 versus 37,600 ± 13,000, respectively, *p* = 0.042). Additionally, there was a 38% decrease in CD8^+^ BT_RM_ in miR-155 inhibitor-treated mice compared to LNA scramble-treated mice (16,800 ± 5800 versus 26,900 ± 11,100, respectively, *p* = 0.035). No significant changes were seen in other cell populations (Fig. 5). Analysis of male and female mice showed a significant difference only in CD11b^hi^CD45^hi^ cells in scramble-treated mice (68,200 ± 14,000 for males versus 33,900 ± 6400 for females). Collectively, these data suggest that miR-155 is required for optimal accumulation of CD8^+^ BT_RM_ following neuroinvasive bacterial infection.

**Fig. 6:**
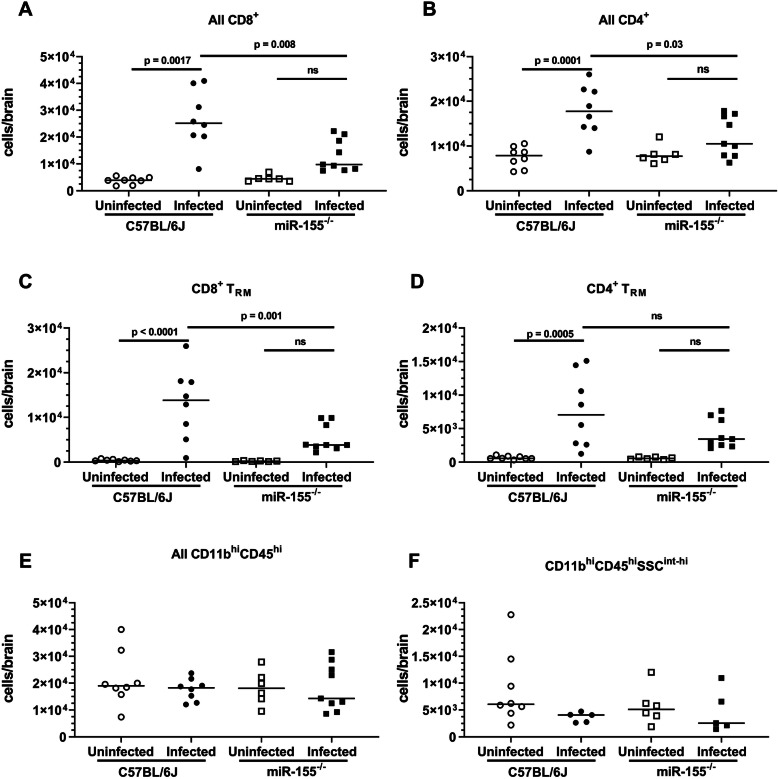
miR-155^−/−^ mice display fewer CD8^+^ BT_RM_ 28d p.i. than do C57BL6/J mice. Male and female C57BL/6J and miR-155^-/-^ mice were infected with 1.2 × 10^5^ CFU *Lm* EGD, then treated with antibiotics beginning 2d p.i. Uninfected mice did not receive antibiotics. Mice were treated as in Fig. 5a, but without injections of inhibitor or scramble. *n* = 3–5 for each sex, genotype, and treatment. Mice were sacrificed on 28d p.i. Brain leukocytes were analyzed by FACS. Symbols represent individual mice. Significance was calculated via two-way ANOVA followed by Tukey’s multiple comparisons test

To confirm the role of miR-155 as an essential component for CD8^+^ BT_RM_ development, age- and sex-matched C57BL6/J and miR-155^−/−^ mice were infected with *Lm* and treated with antibiotics as above. In these experiments, uninfected mice were not treated with antibiotics to better quantify baseline numbers of cells with no stimulation in each genotype. Mice were sacrificed at 28d p.i. and brain leukocytes were analyzed by FACS. At 28d p.i., CD3^+^CD8^+^ and CD3^+^CD4^+^ cell populations were decreased in miR-155^−/−^ mice compared with infected C57BL/6J mice (13,200 ± 6000 versus 26,500 ± 10,900 for miR-155^−/−^ and C57BL/6J, respectively (CD3^+^CD8^+^); 12,100 ± 4500 versus 17,900 ± 5600 (CD3^+^CD4^+^)) (Fig. 6a, b). Notably, numbers of CD8^+^ BT_RM_ in infected miR-155^−/−^ mice were significantly reduced compared to infected C57BL/6 mice (5400 ± 3100 versus 13,000 ± 8000 for miR-155^−/−^ and C57BL/6J, respectively) (Fig. 6c). Expressions of CD49a, Ly6C, PD1, and CD11b were not different between the genotypes (data not shown). In accord with results from mice treated with miR-155 inhibitor, numbers of CD4^+^ BT_RM_ were not different in infected miR-155^−/−^ mice compared with infected C57BL/6J mice (Fig. 6d). Infection of miR-155^−/−^ mice also resulted in non-statistically significant increases in numbers of CD8^+^ and CD4^+^ BT_RM_ in in miR-155^−/−^ mice over uninfected miR-155^−/−^ controls (Fig. 6d). In contrast to CD8^+^ T lymphocytes and CD8^+^ *T*_RM_, numbers of CD45^hi^CD11b^+^ myeloid cells 28d p.i. were not different from uninfected mice in either genotype (Fig. 6e, f), suggesting that these cell populations had returned to their pre-infection level. No differences were observed between male and female mice in any leukocyte populations. Additionally, body weights of miR-155^−/−^ and C57BL/6J mice did not differ at baseline or after infection (data not shown), and mutated mice had no developmental or phenotypic abnormalities at baseline. Similarly, no differences were observed when sexes were considered separately.

## Discussion

CD8^+^ T_RM_ are long-lived T lymphocytes recruited into the CNS in response to infection [11, 41]. They are uniquely positioned to respond rapidly to re-infection or re-emergence of the pathogen, but also can inflict neurological damage through chronic immune activation [14, 47]. Results presented here show that CD8^+^ *T*_RM_ accumulate in the brain after neuroinvasive *Lm* infection. This has not been shown previously in models of neuroinvasive bacterial infection, but is known to occur after viral and protozoal infection of the brain, and recently also by peripheral bacterial and viral infections using a prime-boost strategy [10, 12]. Moreover, data presented here show accumulation of CD8^+^ *T*_RM_ in the brain is reduced in the absence of miR-155. Interestingly, numbers of CD4^+^ *T*_RM_ and CD11b^+^ myeloid cells were not significantly affected, supporting the concept that CD8^+^ BT_RM_ were specifically affected by knock-down of miR-155 [33]. A potential mechanism by which inhibition or deletion of miR-155 decreases CD8^+^ BT_RM_ populations is via the transcription factor, T-bet. T-bet has been reported to be required for formation of CD8^+^ effector and memory T lymphocytes [37, 48]. It is downregulated by SHIP1, which is itself downregulated by miR-155 [36].

The phenotype of *Lm*-induced CD8^+^ BT_RM_ suggests that they are likely to have the potential for pro-inflammatory activity. For example, expression of CD69 and CD103 in particular matches the phenotype of *T*. *gondii*-induced CD8^+^ BT_RM_, which had greater production of TNFα and IFNγ after stimulus than did CD103^−^ BT_RM_ [10]. Additionally, the phenotype of the CD8^+^ BT_RM_ shown here is highly similar to recently reported peripherally induced CD8^+^ BT_RM_ identified in mice that were primed with ovalbumin-loaded dendritic cells, then infected with recombinant ovalbumin-expressing ∆ActA *Lm* [13]. Infection-induced BT_RM_ provide rapid protection against reinfection by the same pathogen, or reactivation of latent infection [10, 13, 34]. In addition, recent data show that CD8^+^ BT_RM_ induced by neurotropic viral infection can inflict neurological damage by secreting IFN-γ, activating microglia, and causing neuronal apoptosis [14, 49, 50]. With the advent of antimicrobial therapy, recurrent CNS infection by the same bacterial pathogen is uncommon outside specific situations such as cerebrospinal fluid leak [51]. Even though BT_RM_ induced by bacterial infections may not have a role suppressing latent infection, they likely have the potential to damage the host similar to BT_RM_ elicited by other pathogens or via bystander activation [52].

miR-155 contributes to inflammatory processes and pathology in the CNS in multiple parenchymal cell types including microglia, astrocytes, and endothelial cells, as well as via influxing leukocytes [23, 53, 54]. Inhibiting miR-155 expression in noninfectious challenges such as stroke, brain trauma, and experimental autoimmune encephalitis decreases brain inflammation, possibly via SOCS1 and SHIP-1 [55–57]. Our prior work in *Lm*-infected miR-155^−/−^ mice showed lack of miR-155 suppressed acute inflammatory responses, as infected miR-155^−/−^ mice had muted IFN-γ activation and M1 polarization of microglia, as well as reduced influxes of bone marrow-derived cells compared with non-mutated mice [23]. Results presented here show that injection of miR-155 inhibitor produced less robust reduction of infection-induced brain inflammatory responses than were found in miR-155^−/−^ mice. In particular, the miR-155 inhibitor did not appear to dampen microglial activation 9d after infection. There are several potential reasons for this. First, the injected inhibitor has to be taken up into cells before it can show an effect, and this step likely varies among cell types and tissues. In contrast, in mutated mice, every cell lacks the ability to produce miR-155. Second, it is doubtful that the inhibitor crosses the blood-brain barrier and therefore is not directly accessible to microglia. To overcome this, others have injected similar miR inhibitors directly into the cerebral ventricles [58, 59]. Third, the miR-155 inhibitor was not administered until 2d p.i. when the host response was already well underway. This was done purposefully to mimic a clinical scenario of initiating treatment in a patient with bacterial CNS infection at the same time that antibiotics were also started. It is highly likely that injecting the inhibitor prior to infection would have produced more robust results, but this option is not available to treating clinicians. Nevertheless, a reduction in numbers of BT_RM_ could be a new means for dampening long-term brain inflammation.

In addition to ameliorating inflammatory pathology, blocking miR-155 during an infective process can inhibit host defenses thereby increasing the burden of infecting microbes. This is an important consideration when suppressing inflammatory responses in the setting of infection as shown in models of Herpes Simplex encephalitis and cerebral malaria [35, 53]. We observed that infected mice given a miR-155 inhibitor in combination with antibiotics lost slightly less weight over the first 7 days of infection than did infected LNA scramble-treated mice, and no mortality was seen by 7d p.i. in either group. This, as well as the finding that bacterial loads in the brain, liver, and spleens at 3d p.i. were not different between the two groups, suggests that the inhibitor was well-tolerated and that knock-down of miR-155 did not adversely affect bacterial elimination.

Analysis of gene expression in bone marrow-derived CD45^hi^ cells collected from the brains of *Lm*-infected mice at 9d p.i. was consistent with an inhibitory effect on T lymphocytes and more specifically on developing CD8^+^ T_RM_ cells that had entered the CNS. Examples of these are the transcription factors *Runx3* and *Nfatc2*. *Runx3* is a transcription factor that is critical to the development of CD8^+^ T_RM_ populations [13, 60, 61]. Lowered expression of *Runx3* can increase apoptosis and thereby reduce CD8^+^ *T*_RM_ populations. In a similar manner, *Nfatc2* (also known as *Nfat1*) is required for T lymphocyte activation and differentiation. *Nfatc2* appears to enhance production of IFNγ, possibly through modulation of T-bet [62]. Other notable changes included reduced expression of *Bcap31*, which encodes a membrane protein in the endoplasmic reticulum that is essential for T lymphocyte activation [63], and increased expression of *Ifngr2*, suggesting acquisition of IFNγ responsiveness and impaired Th1 function [64]. These findings support the concept that the miR-155 inhibitor had a suppressive effect on development and function of activated lymphocytes and BT_RM_.

In contrast with suppressive effects on lymphocyte development and function, genes associated with myeloid cells such as *Camp*, *S100a8*, and *S100a9* were strongly upregulated. Analysis by flow cytometry of sorted cells used for gene expression measurements did not detect increased numbers of myeloid cells in the brain, suggesting that these findings were indeed due to increased gene expression rather than population shifts. These results suggest that the miR-155 inhibitor activated phagocytes, perhaps through upregulated TLR signaling in these cells via downregulation of its direct target, Socs1 [65]. Microglial gene expression, however, was not significantly changed by the peripherally injected miR-155 inhibitor. These results indicate that the effects of the inhibitor on CD8^+^ BT_RM_ were largely due to inhibition of miR-155 expression in peripheral CD8^+^ T lymphocytes. These cells subsequently migrated into the brain.

One of the major limitations of these studies is that specific mechanism and effects of *Lm*-induced CD8^+^ BT_RM_ on cognitive function were not investigated. If *Lm*-induced BT_RM_ behave similarly to other BT_RM_, then limiting the number of BT_RM_ could ameliorate post-infective cognitive decline [14]. However, T cells can also have pro-cognitive effects [66]. Therefore, careful analyses of the extent to which *Lm* infection changes cognitive function in this model and how T lymphocytes are involved in these changes need to be performed. Additionally, analysis of gene expression was performed on the entire population of CD45^hi^ bone marrow-derived leukocytes in the brain. More focused analyses of gene expression in sorted CD3^+^ leukocytes or via single cell sequencing would be more informative of specific changes induced by the miR-155 inhibitor but were beyond the scope of this study. Nonetheless, results presented here provide a strong basis for future inquiries into the role of BT_RM_ and miR-155 into infection-induced cognitive impairment.

## Conclusions

Collectively, these results demonstrate that neuroinvasive *Lm* infection induces accumulation of CD8^+^ *T*_RM_ in the brain. These results, and those from other models, suggest that CD8^+^ BT_RM_ may be found in the brain after CNS infection by other bacterial infections. These are long-lived cells that can cause residual, post-infectious inflammation and contribute to cognitive impairments evident in survivors of CNS infection [14]. Data presented here show for the first time that expression of miR-155 is required for optimal accumulation of CD8^+^ *T*_RM_ cells in the brain and that an exogenous miR-155 inhibitor, given at the same time as antibiotics, decreases the number of eventual CD8^+^ BT_RM_ cells, without substantially affecting other cell populations. Although off-target effects of miR-155 inhibition could produce untoward consequences in a clinical situation, beneficial actions in non-infectious [55–57] as well as infectious situations [35, 53], particularly when pathogen replication is controlled, suggest it could be useful with appropriate targeting. Given the long-lived nature of CD8^+^BT_RM_, it is possible that reducing their accumulation during infection could also reduce their potential for inflicting damage directly, or by a subsequent immune challenge [14, 67]. Further study of CD8^+^ BT_RM_ cells is required to understand their role in post-infectious cognitive outcomes and the degree to which miR-155 inhibition is a viable adjunct to antimicrobial therapy in treatment of neuroinvasive bacterial infection.

## Supplementary information

**Additional file 1: **Supplemental Figure 1. FACS gating strategy for sorting CD45+ magnetically sorted brain cells into bone marrow-derived cells (CD45^hi^) and microglia (CD11b^+^CD45^int^). Male C57BL/6J mice were infected with 2.2 x 10^5^ CFU *Lm*, treated with antibiotics and miR-155 inhibitor or LNA scramble 2d, 4d, 6d, and 8d p.i. The animals were euthanized at 9d p.i. and perfused and the brains removed and digested enzymatically. Brain leukocytes were collected by magnetic sorting with CD45-labeled magnetic beads, then incubated with CD11b and CD45 mAb and sorted by FACS into bone marrow-derived (CD45^hi^) and microglial (CD11b^+^CD45^int^) populations for analysis of gene expression.

**Additional file 2:** Supplemental Figure 2. FACS analysis of CD45^hi^ cells used for analysis of gene expression (A) CD45^hi^CD11b^+^Ly6G^+^ cells as a percent of all CD45^hi^ cells. (B) CD45^hi^CD3^+^ cells as a percent of all CD45^hi^ cells. Results shown are the valued from individual cell pools from 2 mice each, bars are the mean for the sample. Unfortunately, one miR-inhibitor dataset was lost.

**Additional file 3:** Supplemental data file 1.

## Data Availability

Data are available upon reasonable request.

## Abbreviations

BT_RM_: Brain T_RM_
CNS: Central nervous system
d: Days
i.p.: Intraperitoneal
i.v.: Intravenous
*Lm*: *Listeria monocytogenes*
LNA: Locked nucleic acid
p.i.: Post infection
s.c.: Subcutaneous
T_RM_: Tissue-resident memory T cell
